# Identification of a Gene Signature of Cancer-Associated Fibroblasts to Predict Prognosis in Ovarian Cancer

**DOI:** 10.3389/fgene.2022.925231

**Published:** 2022-07-06

**Authors:** Li Zeng, Xuehai Wang, Fengxu Wang, Xinyuan Zhao, Yiqian Ding

**Affiliations:** ^1^ Department of Obstetrics and Gynecology, Nantong Maternal and Child Health Hospital Affiliated to Nantong University, Nantong, China; ^2^ Department of Occupational Medicine and Environmental Toxicology, Nantong Key Laboratory of Environmental Toxicology, School of Public Health, Nantong University, Nantong, China

**Keywords:** ovarian cancer, CAFs, WGCNA, bioinformatics analysis, prognosis

## Abstract

Ovarian cancer (OvCa) is one of the most widespread malignant tumors, which has the highest morbidity and unsatisfactory clinical outcomes among all gynecological malignancies in the world. Previous studies found that cancer-associated fibroblasts (CAFs) play significant roles in tumor growth, progression, and chemoresistance. In the current research, weighted gene co-expression network analysis (WGCNA), univariable COX regression, and the least absolute shrinkage and selection operator (LASSO) analysis were applied to recognize CAF-specific genes. After multiple bioinformatic analyses, four genes (AXL, GPR176, ITGBL1, and TIMP3) were identified as OvCa-specific CAF markers and used to construct the prognostic signature (CAFRS). Furthermore, the specificity of the four genes' expression was further validated at the single-cell level, which was high-selectively expressed in CAFs. In addition, our results showed that CAFRS is an independent significant risk factor affecting the clinical outcomes of OvCa patients. Meanwhile, patients with higher CAFRS were more likely to establish chemoresistance to platinum. Besides, the CAFRS were notably correlated with well-known signal pathways that were related to tumor progression. In summary, our study identifies four CAF-specific genes and constructs a novel prognostic signature, which may provide more insights into precise prognostic assessment in OvCa.

## Introduction

Ovarian cancer (OvCa) is one of the most common malignancies, and its morbidity is the highest among all gynecological malignancies worldwide (Siegel et al., 2022). Due to the fatal aggressiveness, the prognosis of patients with OvCa is quietly poor (Siegel et al., 2022). Increasing numbers of studies report that cancer-associated fibroblasts (CAFs), which are highly versatile, plastic and resilient in primary and metastatic tumors, actively participate in tumor progression based on complicated interactions with other cell types in the tumor microenvironment (TME) (Chen et al., 2021; Czekay et al., 2022). For example, Silvia *et al.* found that diverse CAF subpopulations could promote the growth of cholangiocarcinoma at the single-cell level (Affo et al., 2021). In addition, the cellular crosstalk mediated by CAFs could also support the progression of hepatocellular carcinoma (Song et al., 2021). Collectively, the significant roles of CAFs in mediating oncogenesis and progression of tumors have been preliminarily concluded, suggesting potential targets of them for novel therapies in the future. However, there are few studies regarding CAFs in OvCa.

Increasing numbers of evidence suggested that recognized markers, such as α-smooth muscle actin and fibroblast activation protein α, are carried on CAFs (Nurmik et al., 2020). However, different cancer tissues have specific CAF genes (Biffi and Tuveson, 2021). Thus, using these identified markers may lead to ineluctable deviations in the assessment of CAFs in TME. Single-cell transcriptome analysis can reveal the cellular diversity in TME, such as the sub-populations of CAFs in cholangiocarcinoma (Affo et al., 2021). Although single-cell RNA-sequencing analysis can identify cellular states and their specific genes, the number of cells in tumor tissues is limited and the source of cases is heterogeneous, which may lead to bias. Despite the expression profiles of large cases provided by RNA-sequencing or microarray technologies, the complexity of cellular states and cell subtypes in a tissue mixture may also lead to bias.

Considering the advantages and limitations of single-cell sequencing and RNA sequencing or microarray technology, we firstly assessed the infiltration status of the stroma and CAFs using published algorithms that assess the abundance or levels of infiltration cell populations according to the expression values of some well-known signatures. Then, specific CAF genes were identified using weighted co-expression network analysis (WGCNA) by linking the fraction of CAFs with the relative gene module, and the clinical value of the gene signature was further explored and verified in many other microarray datasets. Finally, the expression specificity of gene signatures of OvCa CAFs was further verified in a single-cell transcriptome dataset.

## Materials and Methods

### Download and Analysis of Public Datasets

The Gene Expression Omnibus (GEO) portal (https://www.ncbi.nlm.nih.gov/geo/) and UCSC Xena website (https://xenabrowser.net/datapages/) were used to obtain gene expression profiles of OvCa patients. After screening, six OvCa-related datasets, including GSE9891 (Tothill et al., 2008), GSE26712 (Bonome et al., 2008), GSE49997 (Pils et al., 2012), GSE140082 (Kommoss et al., 2017), GSE23603 (Marchion et al., 2011), and TCGA-OV cohort were obtained. GSE9891 was used as a training cohort. And the other five datasets were applied as test cohorts. The robust multi-array average (RMA) algorithm in the affy package was conducted to preprocess the array profiles. After background correction, quantile normalization, and probe summarization, the gene expression profile was generated based on the platform providing gene and probe mappings. Samples with overall survival (OS) above zero day were selected for further analysis. All the details of these datasets are presented in Supplementary Table S1.

### Evaluation of Stromal Cell Populations for Ovarian Cancer Patients

To estimate the fraction of CAFs in the TME of OvCa patients in the GSE9891 cohort, the Estimating the proportion of immune and cancer cells (EPIC) method (Racle et al., 2017) was used to estimate the population abundance of CAFs in the training cohort according to the expression levels of signatures for different cell types. In addition, The ESTIMATE algorithm (Yoshihara et al., 2013), a method using gene expression profiles to estimate the fraction of stromal and immune cells in tumor samples, was also conducted to calculate Stromal Score, ESTIMATE Score, Immune Score, and Tumor Purity.

### Identification of Cancer-Associated Fibroblasts-Specific Markers

Previous studies identified shared gene signatures and biological mechanisms in type 2 diabetes and pancreatic cancer by utilizing the WGCNA method (Hu et al., 2022). To further explore the relationship between gene expression and abundance of CAFs, we performed the WGCNA according to the expression profiles of all genes (17,003 genes) by utilizing the R package WGCNA (Langfelder and Horvath, 2008) and next identified the remarkable gene modules positively correlated with the fraction of CAFs. The idea of a soft threshold is to continually elementize the elements in the Adjacency Matrix through a weight function. And due to the choice of the soft threshold, β, can affect the result of module recognition and the relative network of the random average of each node, there is a scale-free network in which a few nodes exhibit a significantly higher degree than the general point, which is a more stable choice. According to published research (Chen et al., 2020), the WGCNA was performed, and the soft threshold power of β = 4 (scale-free R2 = 0.92) was installed. Genes with gene significance (GS) ≥ 0.5 and module membership (MM) ≥ 0.5 in modules highly associated with CAFs were identified as CAF-related markers and extracted for further study.

### Construction of Prognostic Model Based on Cancer-Associated Fibroblasts-Specific Markers

Next, CAF-specific markers were selected to construct the gene risk score following the criteria. Firstly, genes with GS ≥ 0.5 and MM ≥ 0.5 in the module which had the highest correlation with CAF fraction were selected for further analysis. Then, the univariable COX regression analysis was conducted to extract genes significantly associated with OS (*p*-value < 0.05). Subsequently, the least absolute shrinkage and selection operator (LASSO) regression algorithm was utilized to screen the potential prognostic genes, which were defined as CAF-specific markers. The risk score of the prognostic model based on CAF-specific markers (CAFRS) of patients was calculated based on the linear combination of the expression values of CAF-specific markers multiplied by the corresponding LASSO coefficients. To confirm the role of the risk score in the prediction of prognosis, the OvCa patients were divided into the high- and low-CAFRS groups according to the 50% cutoff of the CAFRS.

### Enrichment Analysis of Gene Functions and Pathways

Gene Ontology (GO) and Kyoto Encyclopedia of Genes and Genomes (KEGG) pathway enrichment analysis were performed by using the R package clusterProfile. The top ten enriched GO and KEGG pathways with the most significant *p*-values were displayed.

### Single-Cell RNA Sequencing Datasets Analysis

To explore whether the gene signature screened by WGCNA was consistent with the cell-specific genes of different cells in OvCa, the single-cell transcriptomes datasets of six ovarian tumor tissue from GSE173682 (Regner et al., 2021) were downloaded. All additional analyses were performed using the Seurat (4.0.4, http://satijalab.org/seurat/) R toolkit (Butler et al., 2018), including quality control and all subsequent analyses.

To eliminate the influence of abnormal cells and technical background noise on downstream analysis, cells were reserved if the expression of mitochondrial genes was greater than 10% or with detected genes less than 200 or greater than 5,000. Finally, a total of 50,502 cells were used for further analysis (GSM5276938: 8,009 cells, GSM5276939: 8,295 cells, GSM5276940: 8,181 cells, GSM5276941: 8,984 cells, GSM5276942: 10,094 cells, GSM5276943: 6,939 cells).

In order to minimize the technical batch effects among individuals and experiments, we used the “RunHarmony” function in R package harmony (Korsunsky et al., 2019) to integrate 50,502 cells from six OvCa patients. The top 4,000 variable genes were used for principal component analysis (PCA) to reduce dimensionality. The dimensionality of the scaled integrated data matrix was further reduced to two-dimensional space based on the first 30 principal components (PCs) and visualized by t-Distributed Stochastic Neighbor Embedding (tSNE). The cell clusters were identified based on a shared nearest neighbor (SNN) modularity optimization-based clustering algorithm with a resolution of 1, and all cells were divided into 27 clusters (Supplementary Figure S1B). In order to recognize the types of these cells, some known markers, such as VWF and PECAM1 for endothelial cells, EPCAM and KRT8 for epithelial cells, COL1A1 and DCN for fibroblasts, CD3E and CD3G for T cells, CD86 and LYZ for macrophages, and CD79A for B cells, were used to verify the annotation of cell types (Supplementary Figure S1C).

### Statistical Analysis

All statistical analyses were handled using R-4.0.4. The significant difference in continuous variables between the two groups was assessed using the Wilcoxon rank-sum test, while categorical variables were compared by the chi-square test. Prognostic values were evaluated using the log-rank test. For all analyses, a two-paired *p*-value < 0.05 was deemed to be statistically significant, and labeled with *p-value < 0.05, **p-value ≤ 0.01, ***p-value ≤ 0.001, and ****p-value ≤ 0.0001.

## Results

### Changing Trends in the Stromal and Cancer-Associated Fibroblasts

**FIGURE 1:**
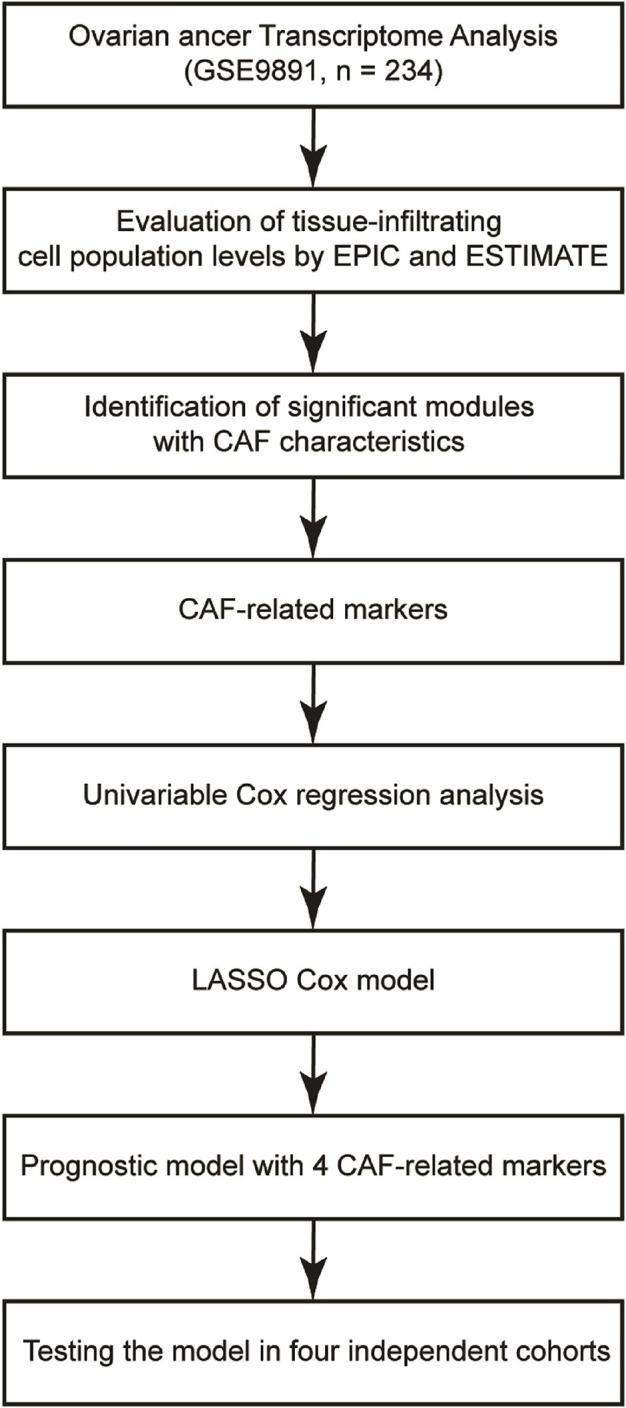
Flow chart of the research. The gene expression profiles of GSE9891 were obtained from the GEO database. ESTIMATE and EPIC algorithms were performed to estimate the infiltration levels of stromal, CAFs and immune cells. WGCNA was used to explore potential genes related to the fraction of CAFs. Univariable Cox regression analysis was performed to screen genes that were notably related to OS. LASSO regression method was used to find out the CAF-specific markers and construct the prognostic model. The prognostic value of the model was validated in the other five independent cohorts. Moreover, the distribution of the CAF-specific markers was displayed at the single-cell level.

**FIGURE 2:**
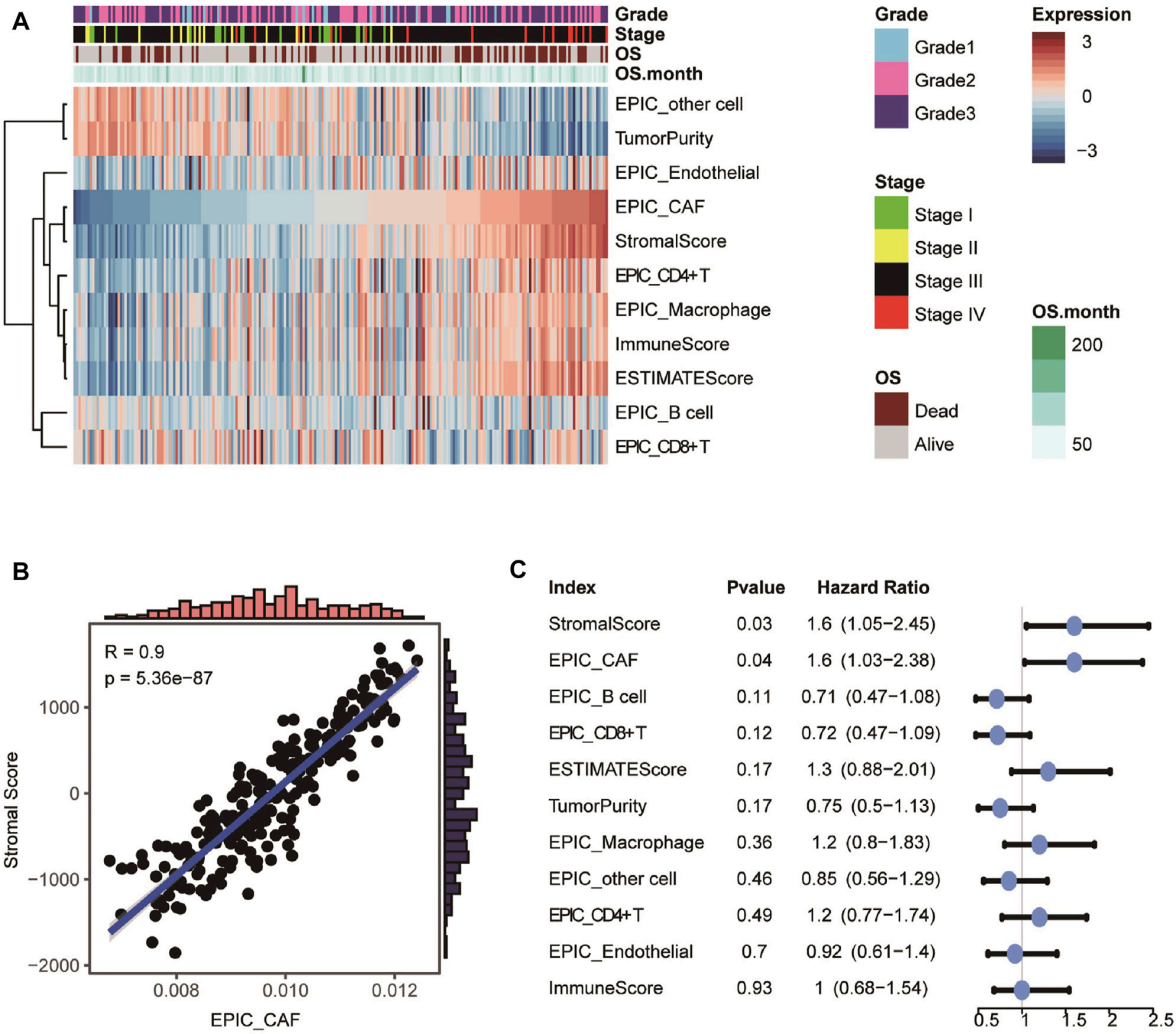
Evaluation and prognostic values of infiltration cell populations. **(A)** Heatmap showing the abundance of tissue-infiltrated cell populations evaluated by ESTIMATE and EPIC algorithm. **(B)** Scatter plot showing the Pearson correlation between CAF fraction evaluated by EPIC and Stromal Score calculated by ESTIMATE. **(C)** Univariable analyses of the abundance of infiltration cell populations with overall survival in GSE9891.

Details of the study’s design were illustrated in Figure 1. Firstly, ESTIMATE and EPIC algorithms were performed to calculate the infiltration of the stromal, CAFs, and immune cells in OvCa. As shown in Figure 2A, OvCa patients with higher tumor stages tended to have a higher proportion of stromal cells and CAFs. As expected, the fractions of CAFs estimated by EPIC for each OvCa patient were significantly positively associated with Stromal Scores (Figure 2B). Meanwhile, high CAF fraction (*p* = 0.03, Hazard Ratio [HR] = 1.6) and Stromal Score (*p* = 0.04, HR = 1.6) were remarkably related to poor OS (Figure 2C), while the relative abundance of immune cell populations and tumor purity were not significantly associated with OS (Figure 2C), indicating the important role of stromal cells, especially the CAFs in the prognosis of OvCa. Overall, these results uncovered the encouraging prognostic values of CAFs in OvCa.

### Identification of Gene Modules and Gene Signatures Correlated to Cancer-Associated Fibroblasts

**FIGURE 3:**
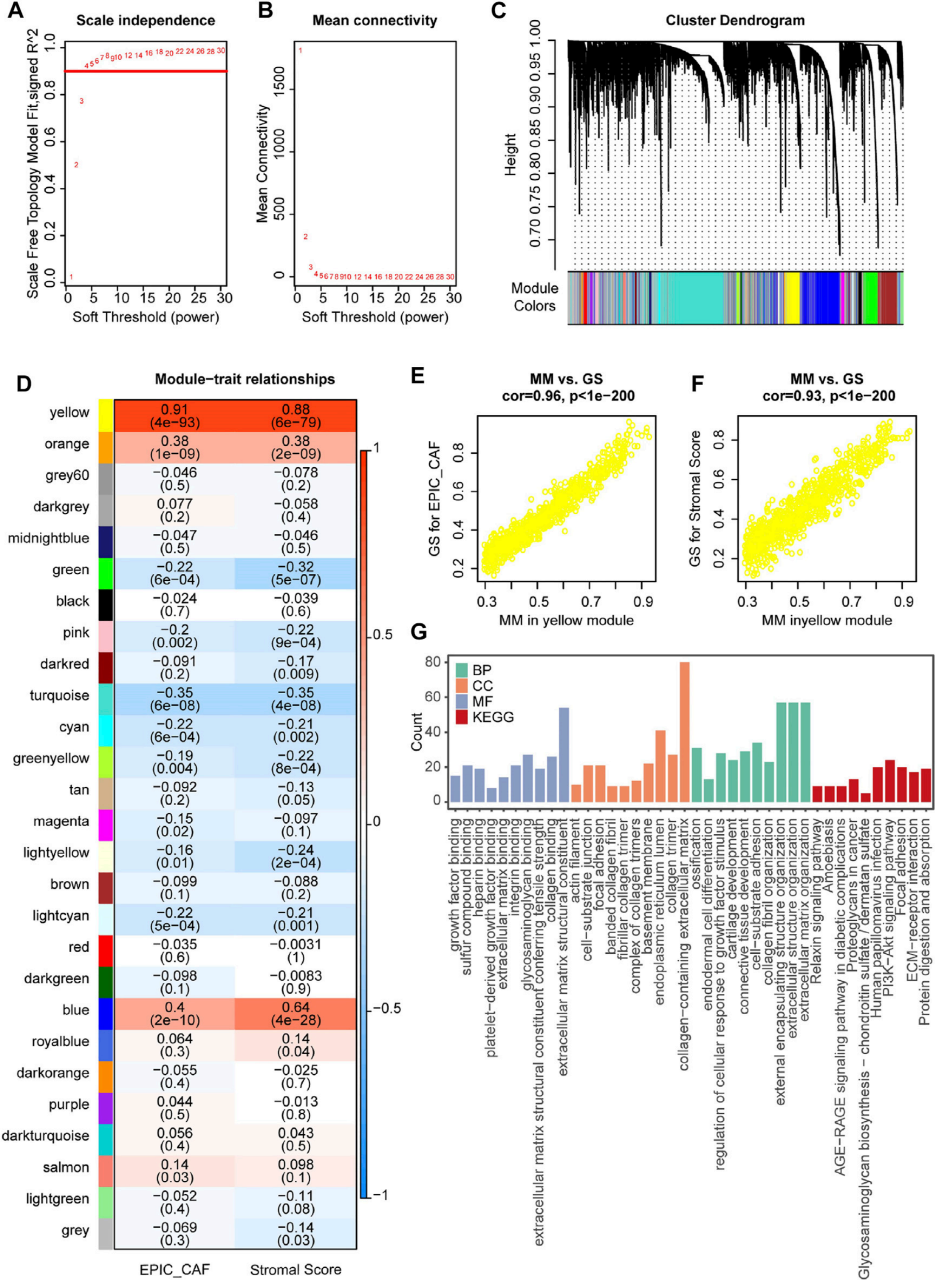
Identification of relevant modules associated with CAF fraction in GSE9891. **(A)** Analysis of the scale-free fitting indices for different soft-thresholding powers (β). **(B)** Mean connectivity analysis of different soft-thresholding powers. **(C)** Clustering dendrograms of genes were based on dissimilarity topological overlap and module colours. As a result, 26 co-expressed modules except the grey module were constructed and labeled with different colours. These modules were arranged from large to small according to the number of genes included. **(D)** Heatmap of the correlation between module eigengenes and CAF or StromalScore of OvCa. The yellow gene module was revealed to exhibit the highest correlation with both CAF fraction and Stomal Score. **(E,F)** Scatter plots showing the relationship between MM and GS in the yellow module. **(G)** GO and KEGG analyses of genes in the yellow module.

**FIGURE 4:**
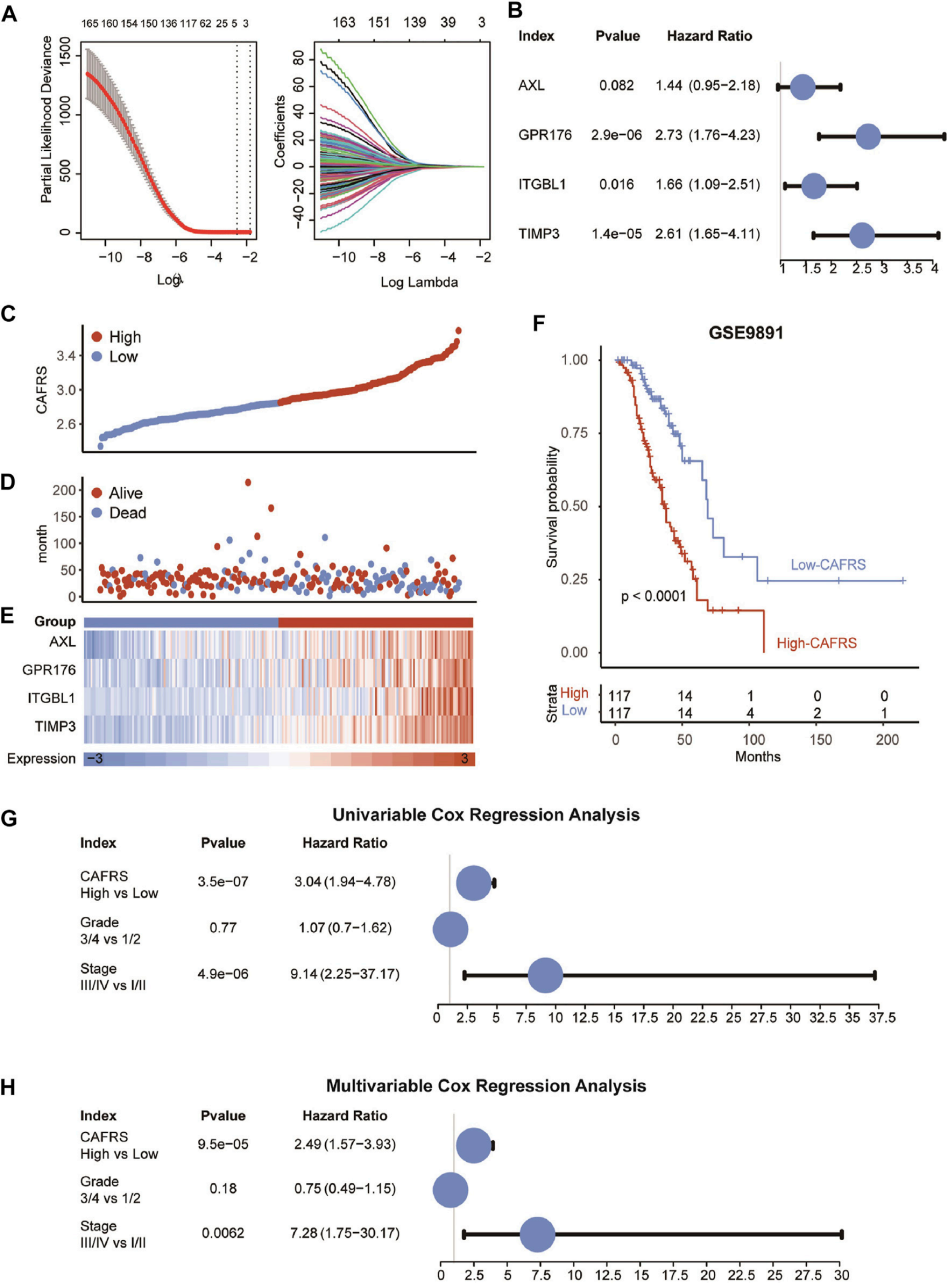
Construction of prognostic model in GSE9891. **(A)** The LASSO coefficient profiles were constructed using CAF-related genes, and the tuning parameter (λ) was calculated based on the minimum criteria for OS with ten-fold cross validation. Four genes were selected according to the best fit profile. **(B)** Univariable analyses of the expression values of the four genes with overall survival in GSE9891. **(C–E)** Distributions of CAFRS, survival status of OvCa patients, and expression profiles of the gene signatures. **(F)** Survival analysis showing the prognostic value of CAFRS in the GSE9891 cohort. **(G–H)** Univariable and multivariable analyses of CAFRS and clinical characteristics in the term of OS in the GSE9891 cohort.

Having observed the prognostic values of CAFs in OvCa, we then constructed a WGCNA by utilizing the R package WGCNA to identify markers associated with CAF fraction in the GSE9891 OvCa cohort. In the current study, the power of β = 4 (scale-free network *R* ^2^ = 0.92) was selected as the soft threshold to ensure a scale-free network (Figures 3A,B). Next, 26 color-coded gene modules except for the gray module were held for further research (Figure 3C). As shown in Figure 4D, the yellow module had the highest correlation with CAFs fraction (R = 0.91, *p* < 0.001) and levels of stromal cells (R = 0.88, *p* < 0.001). The GS and MM values for the yellow module in CAF and StromalScore were displayed in scatter plots (Figures 3E,F). Furthermore, a functional analysis of genes with GS ≥ 0.5 and MM ≥ 0.5 in the yellow module was highly related to extracellular matrix function (Figure 3G), which was produced by CAFs in cancer development (Plikus et al., 2021).

### Identification of Cancer-Associated Fibroblasts-Specific Markers and Constructing a Prognostic Model

The WGCNA and the functional enrichment analysis suggest that genes in the yellow module were positively associated with CAF fraction and functions, then genes with GS ≥ 0.5 and MM ≥ 0.5 in the yellow module were selected as candidate CAF-specific genes. In order to choose genes with significant prognostic values, the univariable COX regression analysis was performed firstly on selected genes that were correlated with OS in OvCa (Figure 4A and Supplementary Table S1). Then, LASSO COX analysis was applied to further reduce the scale of independent prognostic genes to four genes (AXL, GPR176, ITGBL1, and TIMP3) (Supplementary Table S2). The expression of these four genes all behaved with favorable prognosis prediction ability (Figure 4B). Meanwhile, the four genes tended to be highly expressed in patients with higher pathological stages and grades (Supplementary Figures S1A–S1B), consistent with previous results that TIMP3 expression was significantly enhanced in Stage III and Grade 3 (Escalona et al., 2021).

Next, a prognostic model of the four genes was constructed.The CAFRS of patients were calculated according to the combination of the expression levels of these genes multiplied by the corresponding coefficients. The CAFRS of OvCa patients in GSE9891 was further shown in Figures 4C–E. The death cases were focused in the high-CAFRS group, and the surviving cases were centralized in the low-CAFRS group (Figure 4B). Moreover, compared to the low-CAFRS group, four genes in the prognostic signature were higher expressed in the high-CAFRS group. Consistent with the results above, the patients with high CAFRS showed a worse prognosis, compared with patients with low CAFRS (Figure 4F). Furthermore, the prognostic value of CAFRS was explored *via* univariable COX regression analysis of the risk score and multiple clinical characteristics (grade and stage). The univariable COX regression analysis results showed that the improve of CAFRS (HR = 3.04, *p* < 0.001) and stage (stage III and IV, HR = 9.14, *p* < 0.001) were risk factors for OS (Figure 4G). In addition, the results of multivariable COX regression analysis revealed that CAFRS (HR = 2.49, *p* < 0.001) and stage (stage III and IV, HR = 7.28, *p* < 0.001) were independent prognostic factors in OvCa (Figure 4H).

### Independent Prognostic Analysis of Construct the Prognostic Signature in Validation Cohorts

**FIGURE 5:**
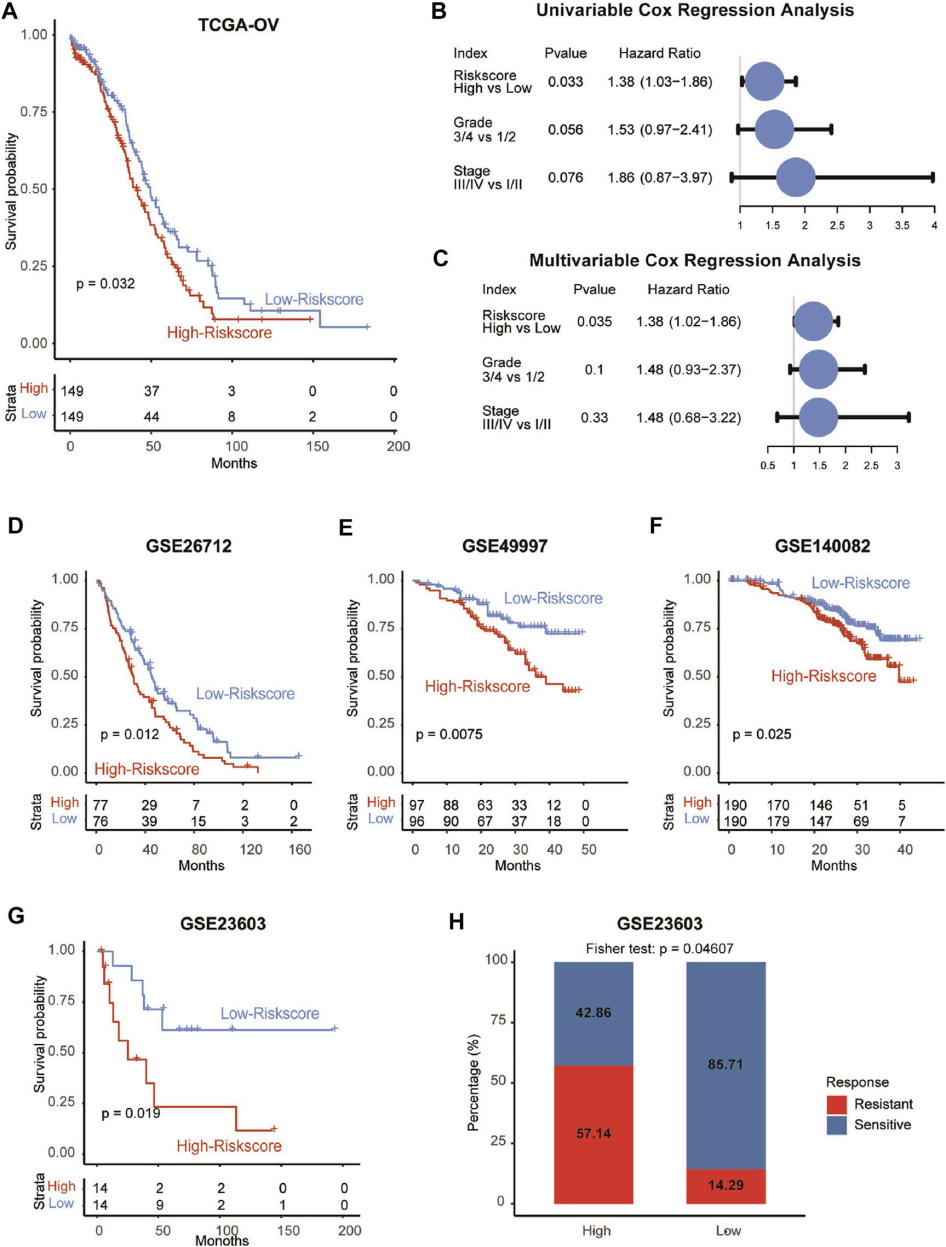
Validation of the prognostic model in five independent cohorts. **(A)** Survival analysis showing the prognostic value of CAFRS in the TCGA-OV cohort. **(B–C)** Univariable and multivariable analyses of CAFRS and clinical characteristics in the term of OS in the TCGA-OV cohort. **(D–G)** Survival analysis showing the prognostic value of CAFRS in GSE26712, GSE49997, GSE140082, and GSE23603 cohorts. **(H)** Barplot showing the percentage of platinum-sensitive and resistant patients in high-CAFRS and low-CAFRS groups.

Given that CAFRS was an independent prognostic factor in OvCa patients in the GSE9891 cohort, we further verified this finding in the TCGA-OV cohort. Consistent with the above results, the four genes tended to be highly expressed in patients with higher clinical stages or grades (Supplementary Figures S2A–S2B). Besides, patients in the high-CAFRS group showed worse prognosis and CAFRS was an independent prognostic factor in the TCGA-OV cohort as well (Figures 5A–C). Considering the importance of genomic mutations in tumor progression, we next performed mutation analysis on the model’s CAF gene to the TCGA dataset. Results showed that there were few mutations in the four genes (Supplementary Figure S3), suggesting that the four genes did not affect the clinical outcomes via genomic mutations.

Consistent with the results found in GSE9891 and TCGA-OV cohort, the prognostic performance of CAFRS was also validated in another four OvCa cohorts (GSE26712, *p* = 0.012; GSE4997, *p* = 0.0075; GSE140082, *p* = 0.025; GSE23603, *p* = 0.019; Figures 5D–G). In addition, OvCa patients with high CAFRS were more likely to exhibit resistance to platinum (*p* = 0.046, Figure 5H), indicating the worse outcome of patients in the high-CAFRS group.

### Distribution and Expression of the Four Cancer-Associated Fibroblasts-Specific Markers at the Single-Cell Level

**FIGURE 6:**
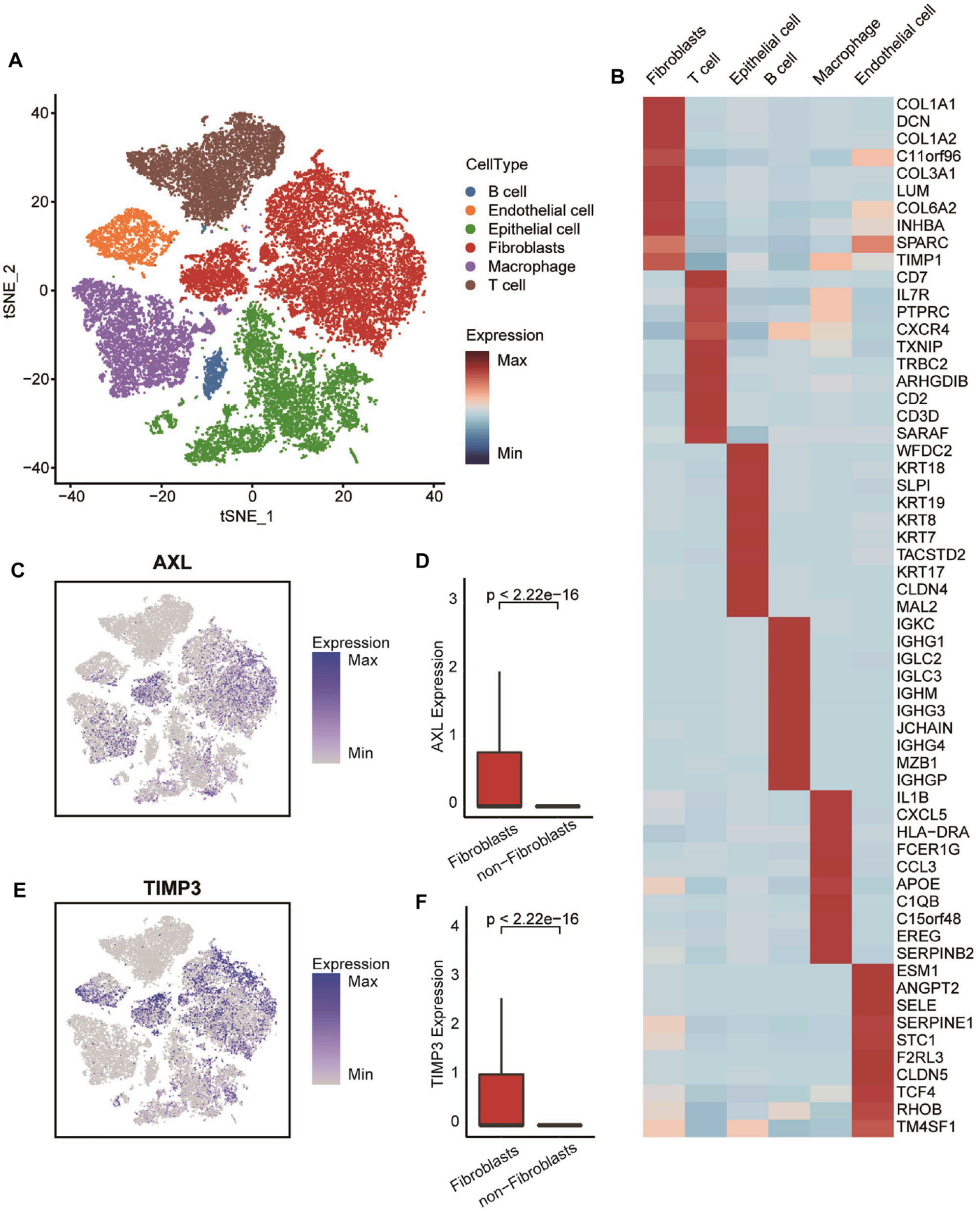
Transcriptomic clustering of six OvCa patients. **(A)** Marker-based cell type identification analysis allowed the prediction of six broad cell types across all profiled single cells. **(B)** Gene expression heatmap of top-10 cell type-specific marker genes as measured by Wilcoxon rank-sum test. **(C,E)** Expression levels of AXL and TIMP3 overlaid on the UMAP representation. **(D,F)** Boxplot showing the expression level of AXL and TIMP3 between fibroblasts and non-fibroblasts. Horizontal lines in the boxplots represent the median, the lower and upper hinges correspond to the first and third quartiles, and the whiskers extend from the hinge up to 1.5 times the interquartile range from the hinge.

In order to confirm the four markers identified by WGCNA based on the expression profiles of tissue mixtures expressed on CAFs specifically, we described the distribution and expression of the four genes in the cell landscape of OvCa at the single-cell level. Single-cell RNA sequencing datasets from six OvCa patients were collected and integrated firstly (Supplementary Figures S3A–S3B). As a result, 50,502 individual cells were passed the quality control criteria (see “Methods” section) and were unsupervised clustered into 27 clusters (Figure 6A). These clusters were explored with unbiased clustering across all cells by PCA and visualized by t-Distributed Stochastic Neighbor Embedding. We annotated the cell type of each cluster with the canonical markers (Figure 6B and Supplementary Figure S1C, Supplementary Table S3), including B cells, endothelial cells, epithelial cells, fibroblasts, macrophages, and T cells. Then, we investigated the distribution and expression levels of four crucial genes. Results showed that all of these genes, especially AXL and TIMP3, were specific highly expressed in fibroblasts (Figures 6C–F and Supplementary Figures S3D–S3E), suggesting that the four genes were OvCa CAF-specific, and the risk score based on them can represent the levels of CAFs fraction.

### Positive Correlation Between Construct the Prognostic Signature and Tumor Progression

**FIGURE 7:**
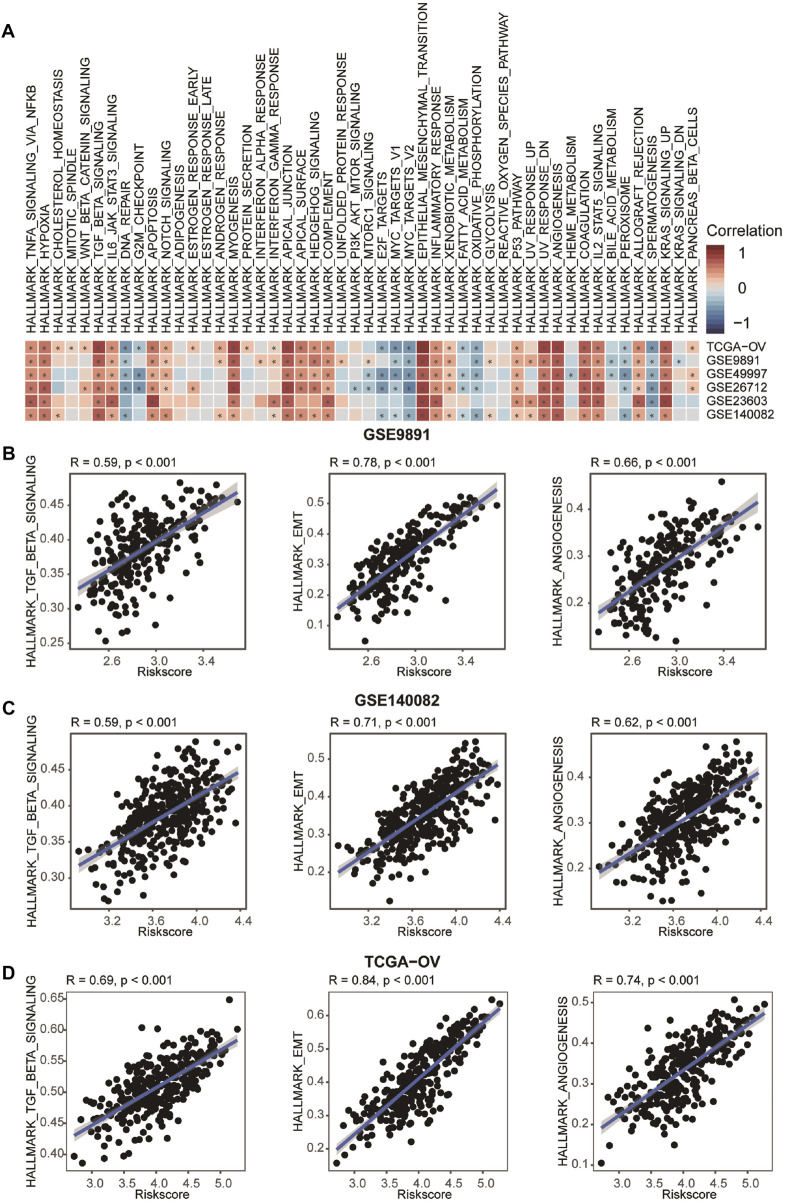
Correlation between CAFRS and HALLMARK. **(A)** Heatmap showing the correlation between CAFRS and HALLMARK scores. The color indicates the correlation coefficient. The asterisks indicate significant differences assessed by Pearson analysis. **(B–D)** Correlation between CAFRS and HALLMARK_TGF_BETA_SIGNALING, HALLMARK_EMT, and HALLMARK_ANGIOGENESIS.

Previous studies reported that CAFs, an important component of TME, play a crucial role in cancer progression (Song et al., 2021). Thus, we further explored the correlation between CAFRS and pathways associated with tumor progression. Results exhibited that the CAFRS were positively related to TGF-β signaling, epithelial-mesenchymal transition and angiogenesis pathways (Figures 7A–D, Supplementary Figures S4A–S4C). Several studies proved that the CAF-mediated TGF-β pathway contributes to cancer progression by regulating many physiological processes, including the promotion of cancer cell proliferation, migration, invasion and metastasis by secreted TGF-β, VEGF and PDGF (Hasegawa et al., 2014; Shi et al., 2020). Conbined with previous studies, our results suggested that CAFRS may reflect the progression of OvCa.

## Discussion

OvCa is one of the most widespread malignancies, which has the highest morbidity and leads to thousands of cancer-related deaths among gynecological malignant tumors in the world (Siegel et al., 2022). Although the underlying mechanisms of OvCa progression are still indistinct, the complex roles of TME in OvCa are gaining attention (Jiang et al., 2020; Song et al., 2020). CAFs, the major cell component of the TME, can lead to the failure of various treatments by exchanging signals with tumor cells during the cancer progression (Errarte et al., 2020). For example, the interaction between CAFs and cancer cells can support glycogenolysis under normoxic conditions and induce phosphorylation and activation of phosphoglucomutase 1, leading to the increased proliferation, invasion, and metastasis of cancer cells (Curtis et al., 2019). Besides, CAFs can influence the actual level of autophagy in OvCa cells through the secretion of pro-inflammatory cytokines and the release of autophagy-derived metabolites and substrates, maintaining the survival and propagation of tumor cells (Thuwajit et al., 2018). Thus, CAFs can be potential targets for novel therapies in the future.

In this research, we reported that the high CAF infiltration in OvCa was related to poor clinical outcomes. And patients with higher pathological stages and grades tended to have higher levels of CAF. Due to the critical function of CAFs in cancer progression and prognostic assessment, the accurate definition of CAF markers within OvCa is significant. Thus, multiple bioinformatics methods were applied in our research to select CAF-specific gene signatures of OvCa and construct the prognostic model. Meanwhile, the independent clinical significance of the CAFRS was validated in the other five datasets, suggesting that CAF-specific genes and the CAFRS could be helpful for individualized prognostic assessment in OvCa.

Several studies have focused on the prognostic values of CAF-related genes in other cancers, a 4-CAF-gene (COL8A1, SPOCK1, AEBP1 and TIMP2) prognostic signature was developed to predict the clinical outcomes and the response to anti-tumor therapies in gastric cancer (Zheng et al., 2021). However, the lack of validation at the single-cell level may lead to bias. In our research, the four OvCa CAF-specific markers (AXL, GPR176, ITGBL1 and TIMP3) were specially expressed in fibroblasts from OvCa at the single-cell level, indicating the more OvCa-specific and accurate of the four genes.

Furthermore, we also found that OvCa patients with high CAFRS were more likely to exhibit resistance to platinum. And the CAFRS were notably correlated with well-known signaling pathways related to tumor progression. Several pieces of evidence reported that CAFs are associated with therapeutic resistance (Kieffer et al., 2020; Zhang et al., 2020; Galbo et al., 2021). The crosstalks between tumor cells and CAFs may lead to the failure of treatment (Fiori et al., 2019). Besides, CAFs can help tumor cells evade immune surveillance (Kieffer et al., 2020) by promoting tumor matrix deposition and remodeling the TME (Sahai et al., 2020), and achieving resistance to treatment finally (Galvani et al., 2020). Moreover, Wang *et al.* proved that the curative effects of immune checkpoint blockade could be reduced by extracellular matrix (Chang, 2019). Due to the unclear sources and functions of CAFs, they will be novel targets for cancer control and overcoming drug-resistance in the future (Sahai et al., 2020).

Although our research recognized and confirmed the clinical significance of OvCa CAF-specific markers and the risk score constructed based on them, further clinical studies are warranted to validate that these signatures can be potential biomarkers or targets of CAFs in OvCa. In addition, given the connections between CFAs and TME, whether the CAF-specific signature could predict the response to immune checkpoint blockade is also worth exploring.

## Conclusion

To sum up, based on multiple bioinformatics analyses, we reported that the enhanced infiltration of fibroblast in OvCa was remarkably associated with worse clinical outcomes. In addition, the 4-gene signature with prognostic significance, which was identified by WGCNA and verified in other independent cohorts and single-cell RNA-sequencing datasets, was significant for future studies on CAFs and shed novel insights into CAFs-target therapy in OvCa.

## Data Availability

The datasets presented in this study can be found in online repositories. The names of the repository/repositories and accession number(s) can be found in the article/Supplementary Material.
