# Supplementary material for: Identification of a Gene Signature of Cancer-Associated Fibroblasts to Predict Prognosis in Ovarian Cancer
Source: Front Genet. 2022 Jul 6;13:925231. doi: 10.3389/fgene.2022.925231 (PMC9298777; doi:10.3389/fgene.2022.925231)
Supplement: Supplementary file 1 [file DataSheet1.DOCX]

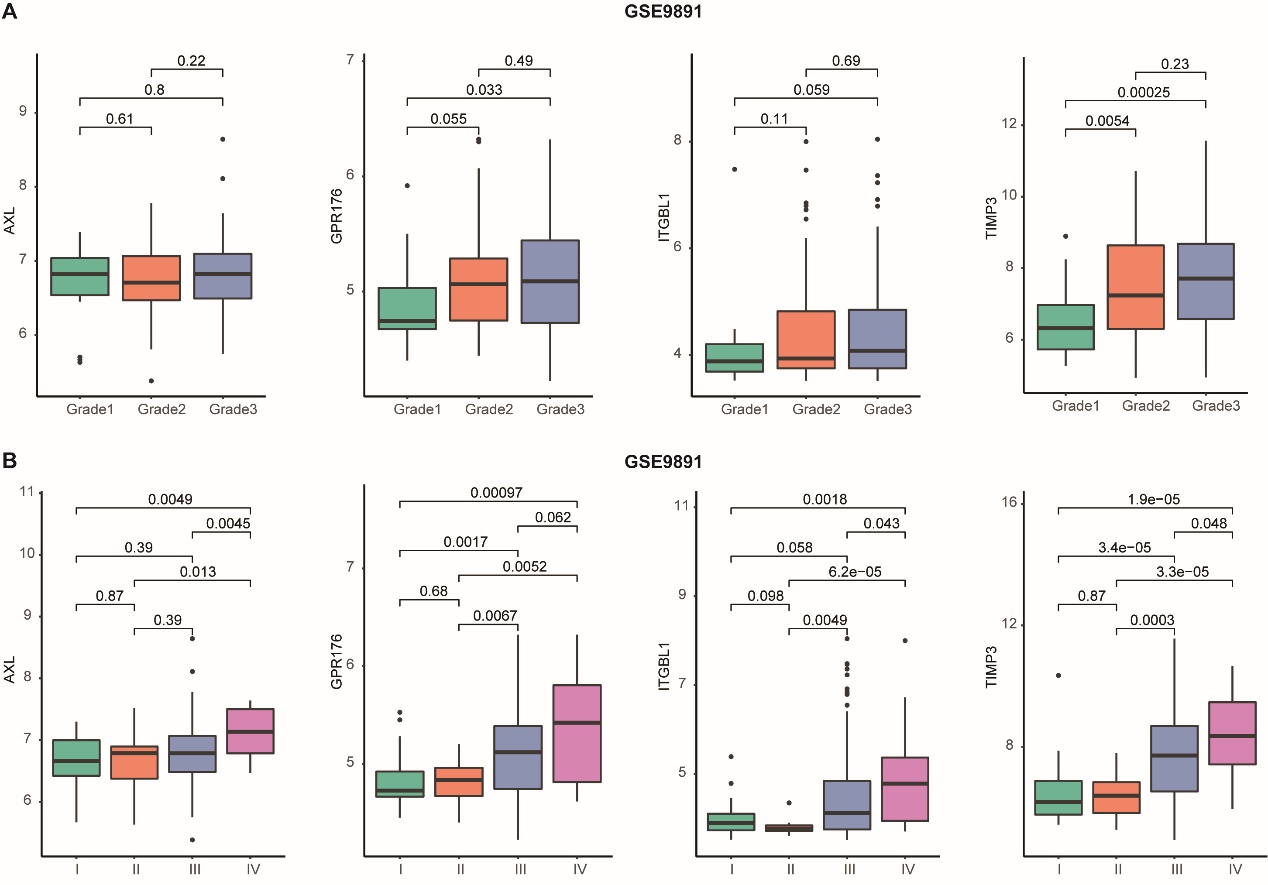


**Supplementary Figure S1. Comparing the expression values of AXL, GPR176, ITGBL1, and TIMP3 among clinicopathological factors in GSE9891.**

(A) Comparing the expression values of AXL, GPR176, ITGBL1, and TIMP3 among Grade. (B) Comparing the expression values of AXL, GPR176, ITGBL1, and TIMP3 among Stage.


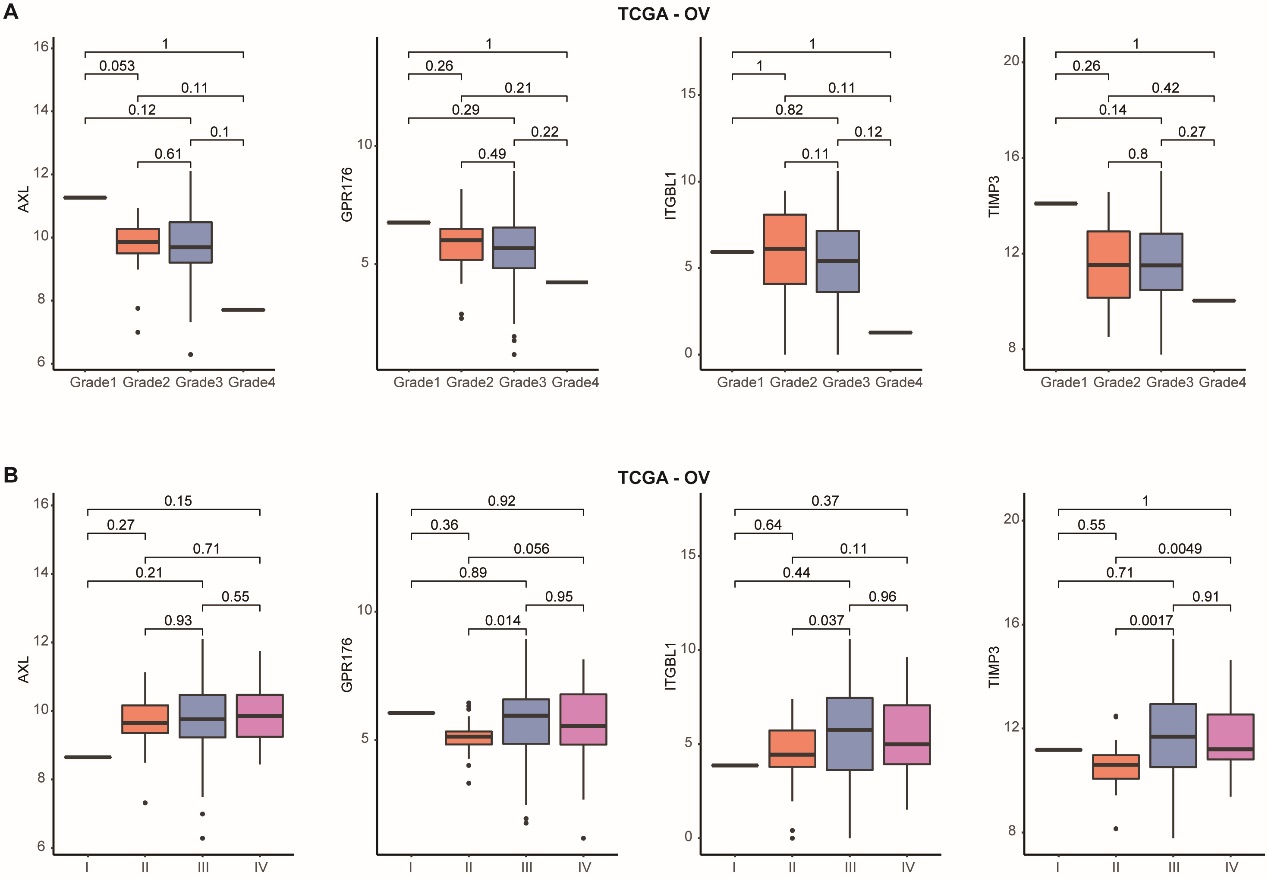


**Supplementary Figure S2. Comparing the expression values of AXL, GPR176, ITGBL1, and TIMP3 among clinicopathological factors in TCGA-OV.**

(A) Comparing the expression values of AXL, GPR176, ITGBL1, and TIMP3 among Grade. (B) Comparing the expression values of AXL, GPR176, ITGBL1, and TIMP3 among Stage.


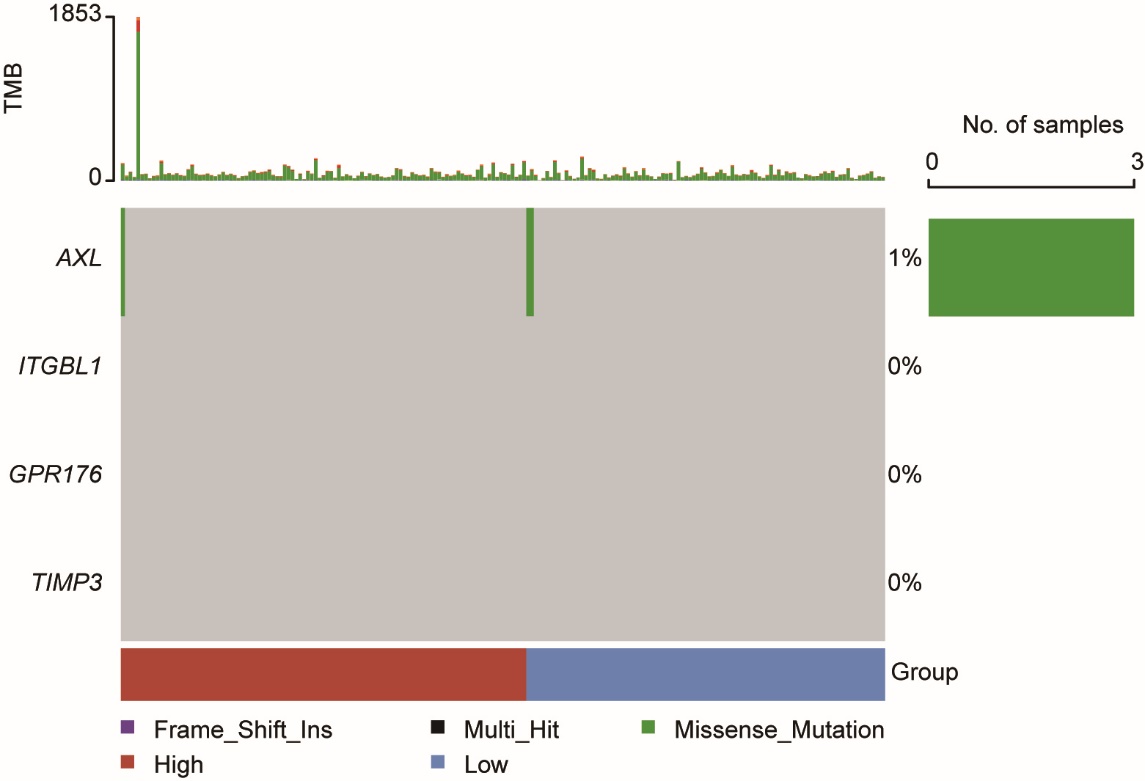


**Supplementary Figure S3. Mutational profiles of model’s CAF genes in the high- and low-CAFRS groups in the TCGA cohort.**


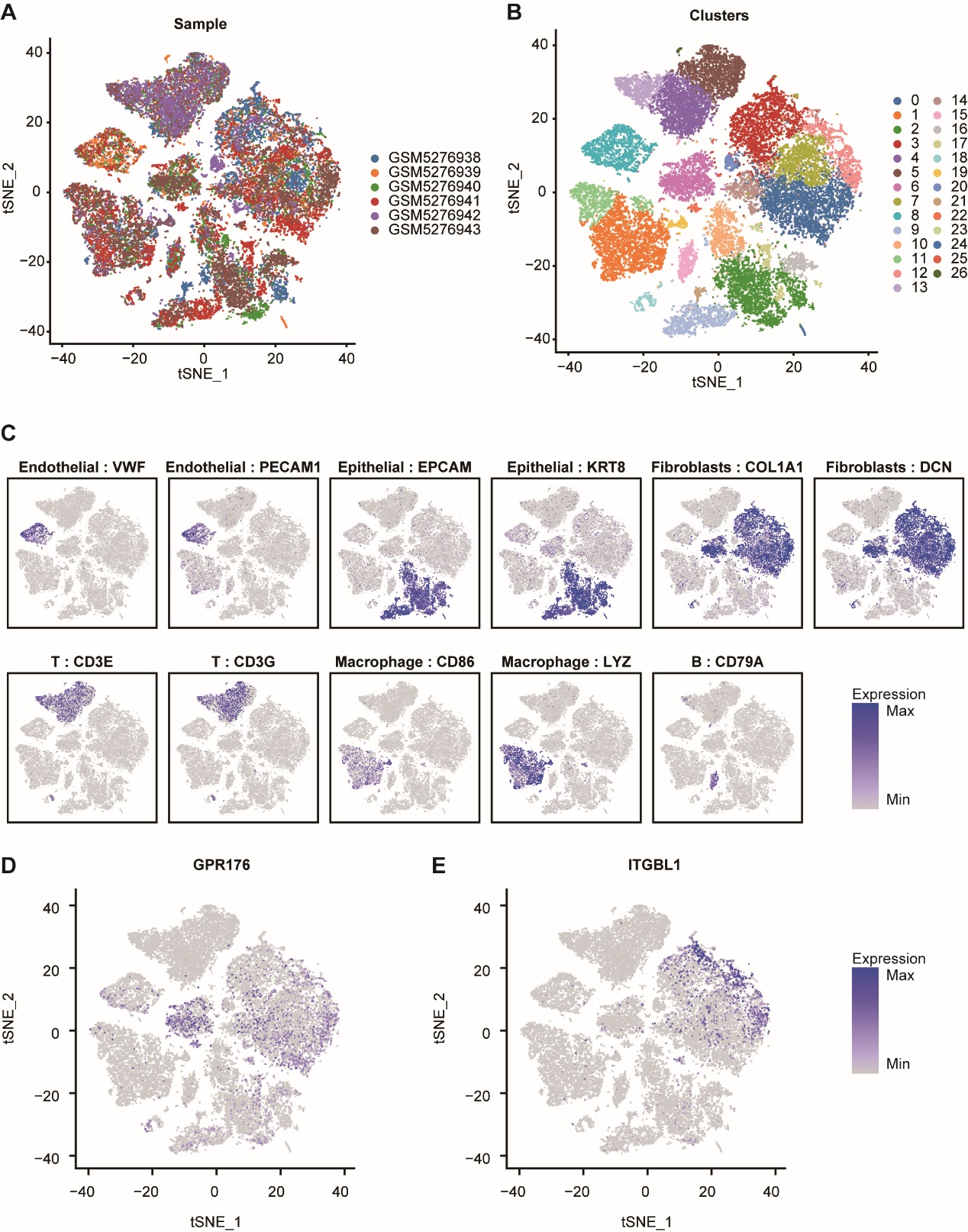


**Supplementary Figure S4. Annotation of scRNA-seq data from OvCa patients.**

(A) t-SNE visualization of 36,851 single cells from six OvCa patients. (B) The unsupervised clustering of 118,565 cells. (C) Expression levels of known markers for cell types overlaid on the t-SNE representation. (D, E) Expression levels of GPR176 and ITGBL1 overlaid on the UMAP representation.


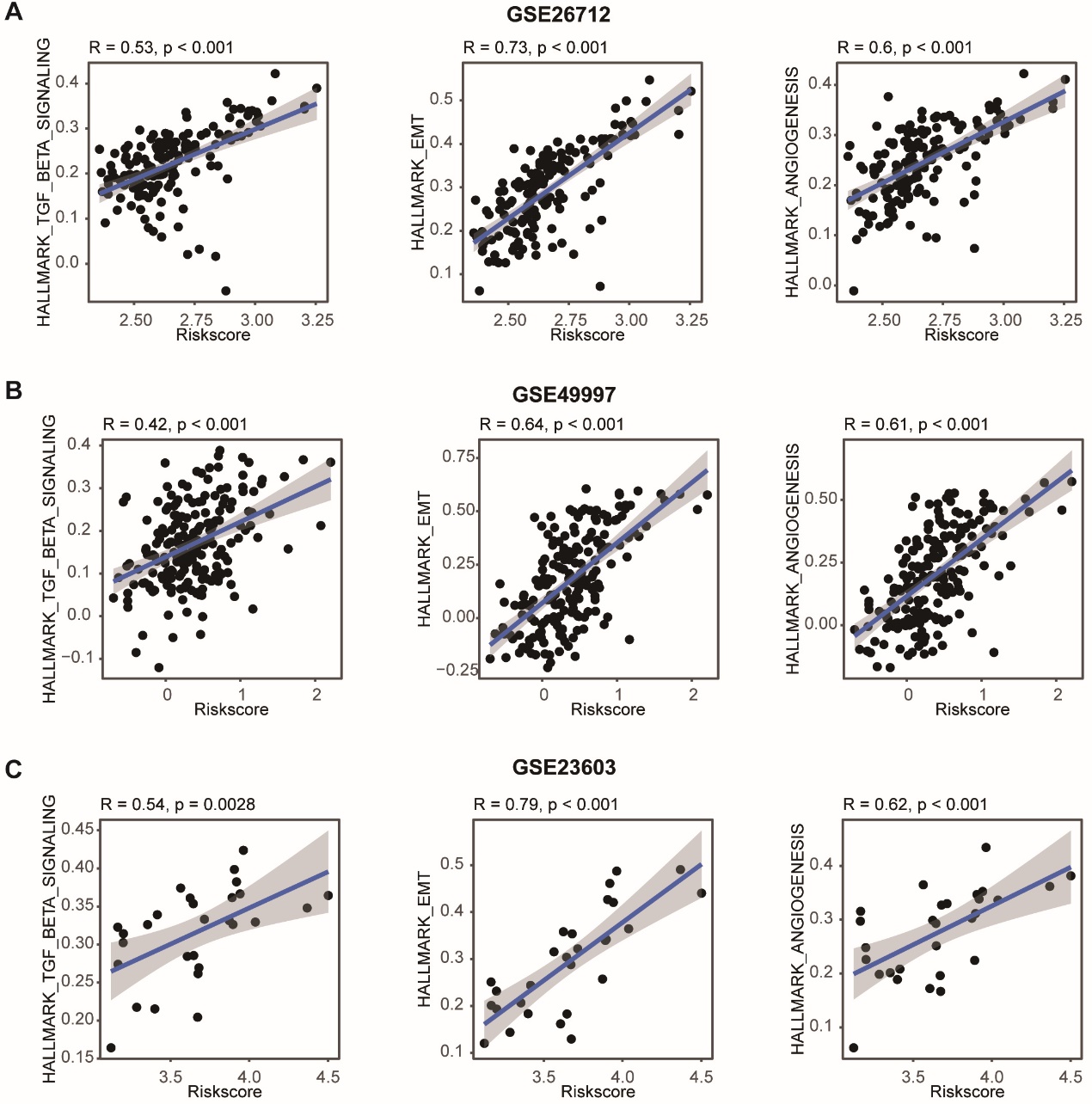


**Supplementary Figure S5. Correlation between CAFRS and HALLMARK.**

(A-C) Correlation between CAFRS and HALLMARK_TGF_BETA_SIGNALING, HALLMARK_EMT, and HALLMARK_ANGIOGENESIS in GSE26712, GSE49997, and GSE23603, respectively.
